# The S-100B level, intracranial pressure, body temperature, and transcranial blood flow velocities predict the outcome of the treatment of severe brain injury

**DOI:** 10.1097/MD.0000000000030348

**Published:** 2022-09-23

**Authors:** Sebastian Dzierzęcki, Mirosław Ząbek, Gabriela Zapolska, Ryszard Tomasiuk

**Affiliations:** a Department of Neurosurgery, Postgraduate Medical Centre, Warsaw, Poland; b Gamma Knife Centre, Brodno Masovian Hospital, Warsaw, Poland; c Clinical Department of Neurosurgery, Central Clinical Hospital of the Ministry of the Interior and Administration, Warsaw, Poland; d Department of Radiology, Brodno Masovian Hospital, Warsaw, Poland; e Kazimierz Pulaski University of Technology and Humanities Radom, Faculty of Medical Sciences and Health Sciences, Radom, Poland.

**Keywords:** biomarkers, brain injury, diagnosis, Doppler, neurosurgery, transcranial

## Abstract

This study evaluates the applicability of S100B levels, mean maximum velocity (*V*_mean_) over time, pulsatility index (PI), intracranial pressure (ICP), and body temperature (*T*) for the prediction of the treatment of patients with traumatic brain injury (TBI). Sixty patients defined by the Glasgow Coma Scale score ≤ 8 were stratified using the Glasgow Coma Scale into 2 groups: favorable (FG: Glasgow Outcome Scale ≥ 4) and unfavorable (UG: Glasgow Outcome Scale < 4). The S100B concentration was at the time of hospital admission. *V*_mean_ was measured using transcranial Doppler. PI was derived from a transcranial Doppler examination. *T* was measured in the temporal artery. The differences in mean between FG and UG were tested using a bootstrap test of 10,000 repetitions with replacement. Changes in S100B, *V*_mean_, PI, ICP, and *T* levels stratified by the group were calculated using the one-way aligned rank transform for nonparametric factorial analysis of variance. The reference ranges for the levels of S100B, *V*_mean_, and PI were 0.05 to 0.23 µg/L, 30.8 to 73.17 cm/s, and 0.62 to 1.13, respectively. Both groups were defined by an increase in *V*_mean_, a decrease in S100B, PI, and ICP levels; and a virtually constant *T*. The unfavorable outcome is defined by significantly higher levels of all parameters, except *T*. A favorable outcome is defined by S100B < 3 mg/L, PI < 2.86, ICP > 25 mm Hg, and *V*_mean_ > 40 cm/s. The relationships provided may serve as indicators of the results of the TBI treatment.

## 1. Introduction

Traumatic brain injury (TBI) is the result of brain damage caused by angular and/or linear acceleration or deceleration forces leading to axonal injury. It manifests itself as neurological, neuropsychological, and psychiatric dysfunction.^1^

In developed countries, the incidence of TBI is 0.2% of the population per year among hospitalized patients.^2^ Every year, almost 2 million people suffer from minor TBI worldwide, leading to temporary disability.^3^ However, in the United States and Europe, almost 20 and 15 people per 100,000, respectively, die from TBI.^2^

The age distribution for TBI is as follows: 15 to 30, 30 to 60, and >60 years.^2^ TBI is most often seen between 15 and 30 years of age, while it is less common in the age group of 30 to 60 years. In the broad spectrum of TBI, it is possible to distinguish craniocerebral injury (CBI), a condition that is often interchangeably used with brain injury.^4^

CBI most frequently affects 2 age groups: 15 to 24 and >75 years.^5^ Although it is internationally prevalent at 1.3 and 2.0 cases per 100,000 in North America and Europe, it oscillates around 0.07 cases per 100,000 in Poland. However, the high frequency of CBI imposes severe economic stress on health and insurance services due to costly and complicated treatment and rehabilitation processes.^6–8^

There are a variety of methods that facilitate the diagnosis of TBI and CBI. They include the Glasgow Coma Scale (GCS), computerized tomography (CT), magnetic resonance imaging (MRI), transcranial Doppler (TCD), and measurements of cerebral perfusion pressure (CPP) and biochemical blood markers.

Therefore, the severity of CBI can be assessed using GCS^9^ for patients whose total score indicates a high level of consciousness. CT and MRI are frequently used in the diagnosis of TBI. CT helps to accurately detect life-threatening and surgically treatable intracranial hemorrhages in patients with TBI and CBI.^10^ However, it provides a moderately valid prognosis.^11^ MRI is the overall superior method for the detection and prognosis of TBI. However, it has a limited ability to detect axonal injury.^12^ TCD^13^ and CPP measurement^14^ are also used as means of diagnosing TBI. TCD is a noninvasive technique that allows real-time monitoring of CPP,^15^ intracranial pressure (ICP),^16^ and cerebral blood flow (CBF).^17^ TCD spectral analysis is also used to determine the maximum systolic velocity, the end-diastolic velocity, and the mean maximum velocity (*V*_mean_) in insonated blood vessels.^18^ The clinical practicality of *V*_mean_ measurement has been confirmed in severe cases of TBI.^19^ CPP was used to assess the pulsatility index (PI) values of the middle cerebral artery.^20^ CBF levels were evaluated using mean blood flow velocity.^21^

To date, a variety of blood biomarkers have been proposed for the diagnosis of acute TBI. They include lactate dehydrogenase,^22^ myelin alkaline protein,^23^ neuron-specific enolase,^24^ creatine kinase, and S100B.^25^ S100B has been shown to play a crucial role in intracellular processes^26^ and induce apoptosis at micromolar concentrations.^27^

Body temperature (*T*) measurement was also considered a diagnostic factor for patients with TBI.^28^ Although brain temperature cannot be reliably predicted from *T*,^29^ some recommend monitoring brain and body temperatures to reduce the risk of temperature-related neuronal damage.^30^

Since the effectiveness of CBI treatment is a derivative of diagnostic quality, there is an ongoing exploration of parameters applicable to a robust, rapid, and error-free prediction of the severity and diagnosis of CBI.^31–34^ Therefore, this study was designed to address the need to build a reliable diagnostic tool for the diagnosis of CBI. The study evaluated the usability of a battery of diagnostic markers, including S100B, *V*_mean_, PI, ICP, and *T*. It was carried out in patients stratified into 2 groups, that is, favorable (FG) and unfavorable (UG) outcomes, defined by the GCS score evaluated 30 days after hospital admission.

## 2. Methods

### 2.1. Study subjects

This study was carried out according to the regulations of the Bioethics Committee of the Warsaw Medical Center for Postgraduate Education. Informed consent was obtained from all subjects or their guardians and, if subjects were under 18 years old, from their parents and/or legal guardians prior to the study.

After admission to the Department of Neurosurgery and Trauma of the Nervous System, the health of the patients was evaluated using the GSC.^35,36^ All of them were subjected to standard diagnostic and therapeutic procedures.^37^ However, those with poor ventilation underwent a gasometric examination to optimize *p*CO_2_ (range: 30–40 mm Hg) and maintain hematocrit and hemoglobin levels at 30% to 40% and 12 to 14 g/dL, respectively.

**Figure 1.**
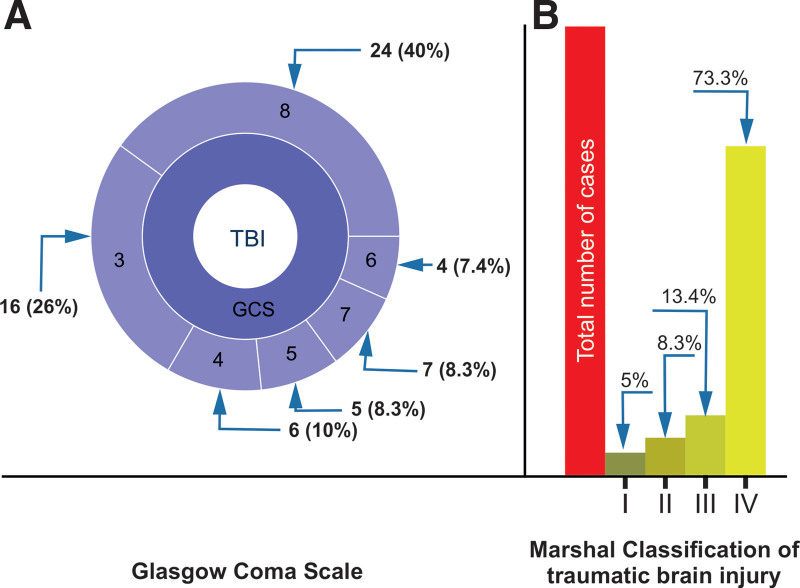
Clinical classification of patients admitted to the Department of Neurosurgery and Trauma of the Nervous System of the Medical Center of Postgraduate Education, Warsaw, Poland. (A) GCS score distribution; (B) MCTC score distribution. Numerals correspond to the number of cases (percentage of cases). GCS = Glasgow Coma Scale, MCTC = Marshall Computed Tomography Classification, TBI = traumatic brain injury.

Only those with a GCS score ≤ 8 were included in the study. Therefore, the study group comprised 60 patients (48 men and 12 women) age range 21 to 75 years; the GCS and the corresponding Marshall scale^38,39^ of the patients included in the study are shown in Figure 1. Study samples comprising FG and UG were obtained by stratification using the Gosling outcome scale (GOS)^40^ and evaluated on the day of hospital discharge. The UG consisted of patients with a GOS score < 4, and the FG consisted of patients with a GOS score ≥ 4.

### 2.2. Parameters and laboratory methods

The parameters studied in this study were measured at 24-hour intervals for 96 hours, 30 days after admission to the Department of Neurosurgery.

S100B concentration was measured in 5 mL of venous blood samples collected from patients at hospital admission. Subsequent blood samples were collected at 24-hour intervals for 96 hours. After clotting and centrifugation for 10 minutes at 1000 revolution per minute, blood samples were stored for further use at –22°C. S100B concentration was measured using a commercially available kit (Liason Sangtec 100). The Sangtec 100 kit uses 3 different monoclonal antibodies (SMST12, SMSK25, and SMSK28) directed against the β-chains of the S100B homodimer and defines a broad diagnostic spectrum between 0.02 and 30.00 µg/L. Protein concentration was measured using a (LIAISON analyzer, Saluggia, Italy) calibrated with a freeze-dried (Sangtec 100 Cal, Dietzenbach, Germany) (low/high) calibrator. The sensitivity threshold for this test was 0.02 µg/L.

*V*_mean_ was measured by subjecting patients to TCD using a Mediasonic Transpect CDS Doppler in power motion mode TCD (PMD/TCD).^41,42^ It was a 2-step procedure: First, the accessible arteries of the brain base were examined through the temporal window, and second, the middle cerebral arteries on the dominant or right side of the extent of the lesion were further analyzed.

PI^43^ was derived from a TCD examination performed using a Mediasonic Transpect CDS Doppler in PMD/TCD. Initially, the brain base arteries accessible through the temporal window were scanned. However, further analysis conducted on the middle cerebral arteries on the dominant or right side of the lesion’s extent was symmetrical.

For each patient, ICP and CPP were measured using implanted microsensor ICP (Codman) sensors. After reset, the ICP sensors were placed in the last stage of surgical removal of the intracranial hematoma or within hours of admission for patients without the characteristics of the intracranial hematoma. All surgical patients underwent osseous dural decompression, which consisted of removing the bone flap and opening the dura mater as wide as possible.

*T* was measured by temporal artery temperature. This method is based on infrared emissions radiating from the skin.^44^ To minimize measurement-induced errors,^45^ each measurement was repeated 3 times and an average of 5 temperature readings was reported. *T* measurements that differed more than 0.5°C were rejected from the mean calculation of *T*.

The self-assessed S100B, *V*_mean_, and PI reference ranges were determined using a group of 40 healthy volunteers, 25 men and 15 women, with an average age of 47.0 ± 14.77 (range: 21–80) years.

### 2.3. Statistical analysis

The normality test was performed using the Shapiro–Wilk^46^ test. Data are expressed as mean ± standard deviation and median with minimum and maximum values. Computation of the rate of change in a parameter was performed using a linear regression model. Differences in the means of the study groups, that is, FG and UG at specific time points, were tested using a bootstrap test for differences in the means of 10,000 repetitions with replacement.^47^ Changes in S100B, *V*_mean_, PI, ICP, and *T* levels stratified by the study group were calculated using the one-way aligned rank transform for the nonparametric factorial analysis of variance technique.^48^ Because of the shortcomings of current statistical methods in handling advanced nonparametric statistics, only the result of one-way nonparametric factorial analysis of variance has been discussed in this study. Patient mortality was taken into account by censoring the number of subjects in a group. The rate of change of a specific parameter was evaluated using the slope of a linear regression model. *P* values < 0.01 were considered statistically significant. All analyses were performed using the R programming language.^49^

## 3. Results

**Figure 2.**
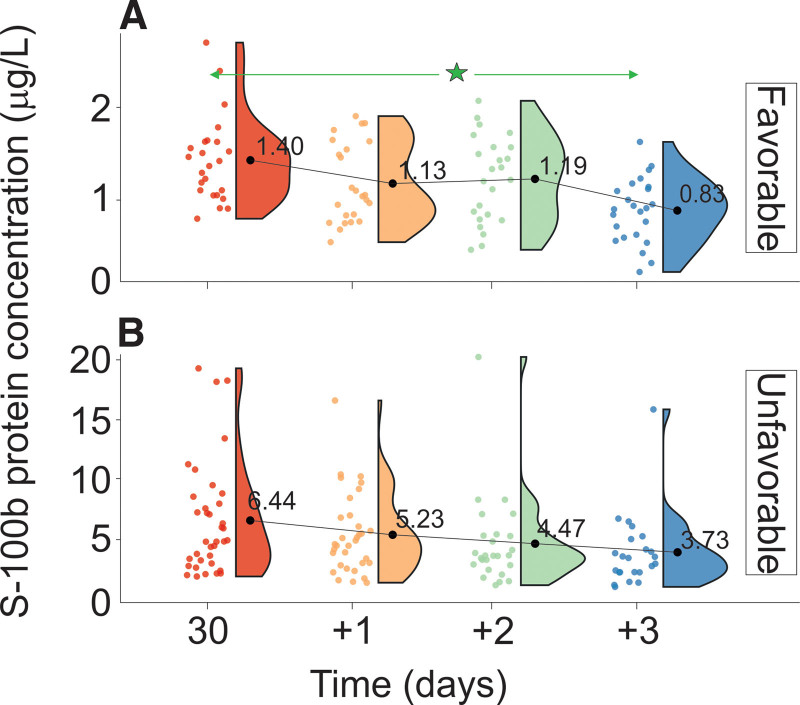
Changes in S100B concentration stratified by GOS levels evaluated on discharge from the Department of Neurosurgery. (A) Favorable outcome group and (B) unfavorable outcome group. The vertical size of the cloud refers to 95% CI at a specific time point. The green arrow shows statistically significant differences in the “favorable” outcome group. GOS = Gosling Outcome Scale. ^*^P < .01.

**Figure 3.**
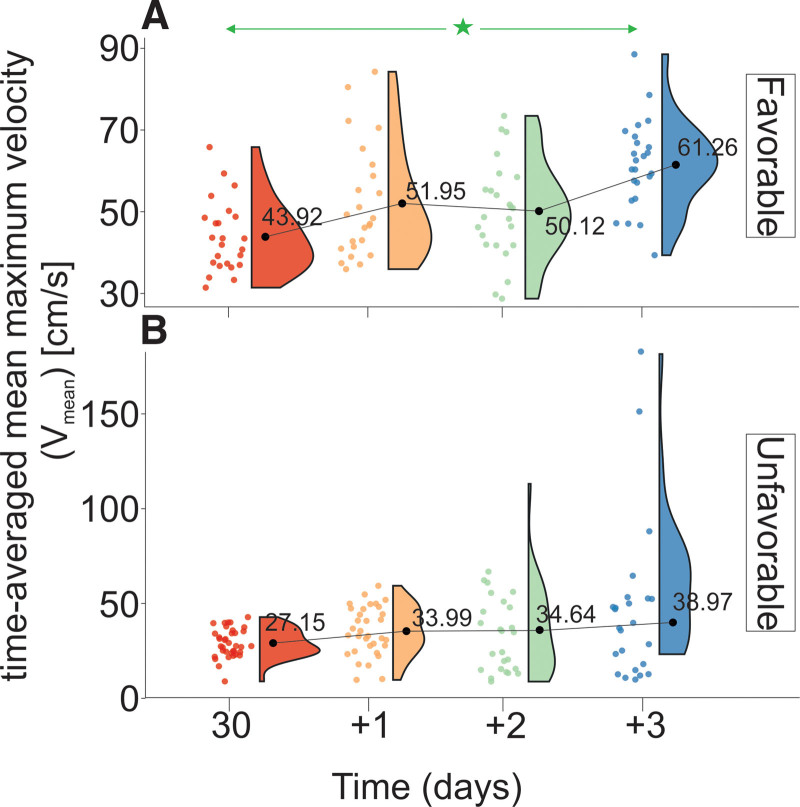
Changes in *V*_mean_ stratified by GOS levels evaluated on discharge from the Department of Neurosurgery. (A) Favorable outcome group and (B) unfavorable outcome group. The vertical size of the cloud refers to 95% CI at a specific time point. The green arrow shows statistically significant differences in the “favorable” outcome group. CI = confidence interval, GOS = Gosling Outcome Scale, *V*_mean_ = time-averaged mean maximum velocity. ^*^P < .01.

**Figure 4.**
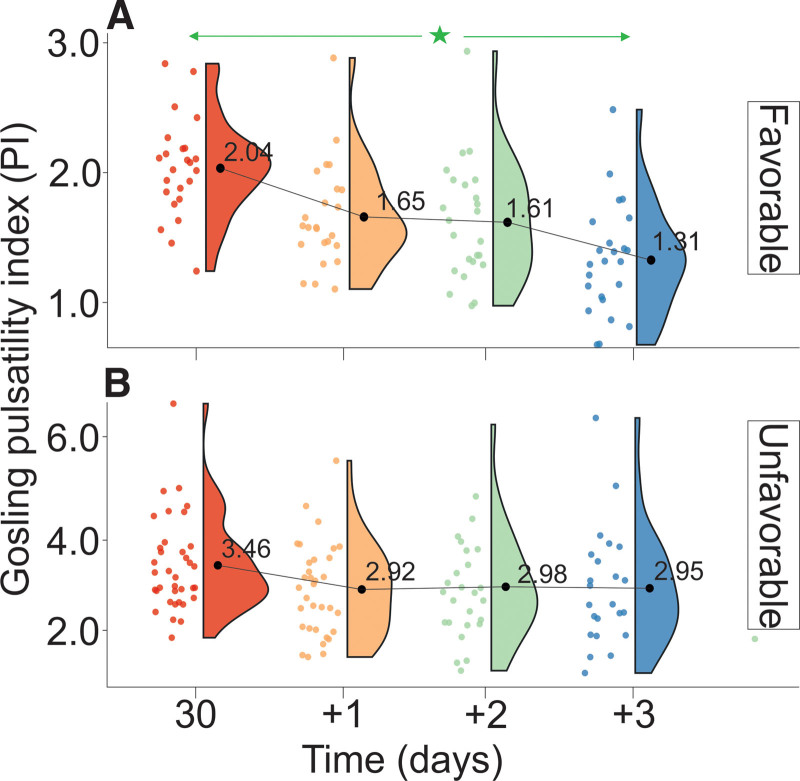
Changes in PI stratified by GOS levels evaluated on discharge from the Department of Neurosurgery. (A) Favorable outcome group and (B) unfavorable outcome group. The vertical size of the cloud refers to 95% CI at a specific time point. The green arrow shows statistically significant differences in the “favorable” outcome group. CI = confidence interval, GOS = Gosling Outcome Scale, PI = pulsatility index. ^*^P < .01.

**Figure 5.**
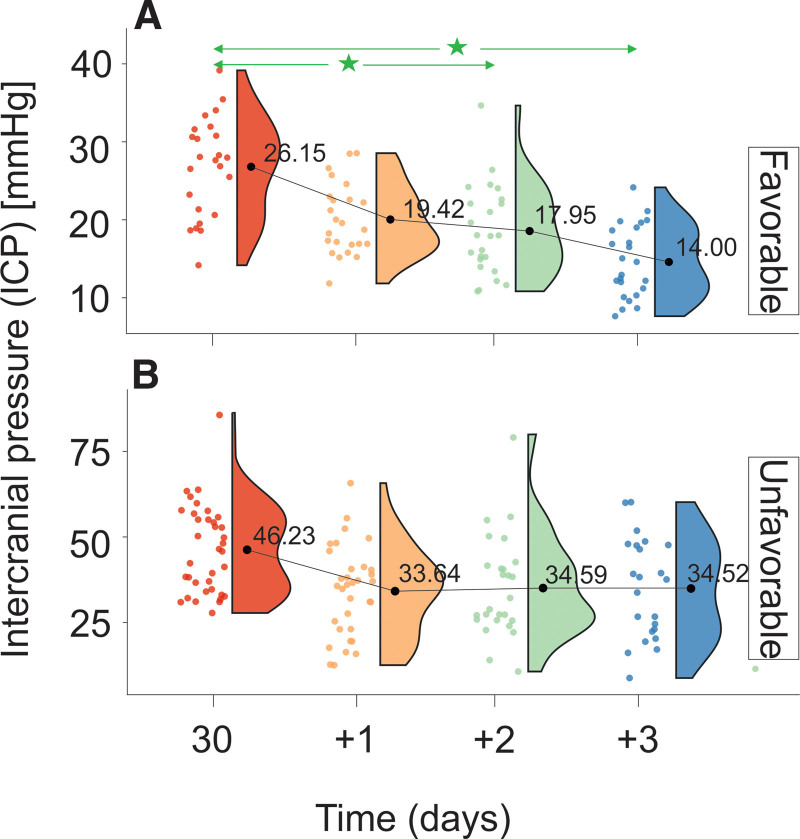
Changes in ICP stratified by GOS levels evaluated on discharge from the Department of Neurosurgery. (A) Favorable outcome group and (B) unfavorable outcome group. The vertical size of a cloud refers to 95% CI at a specific time point. The green arrow shows statistically significant differences in the “favorable” outcome group. CI = confidence interval, GOS = Gosling Outcome Scale, ICP = intracranial pressure. ^*^P < .01.

**Figure 6.**
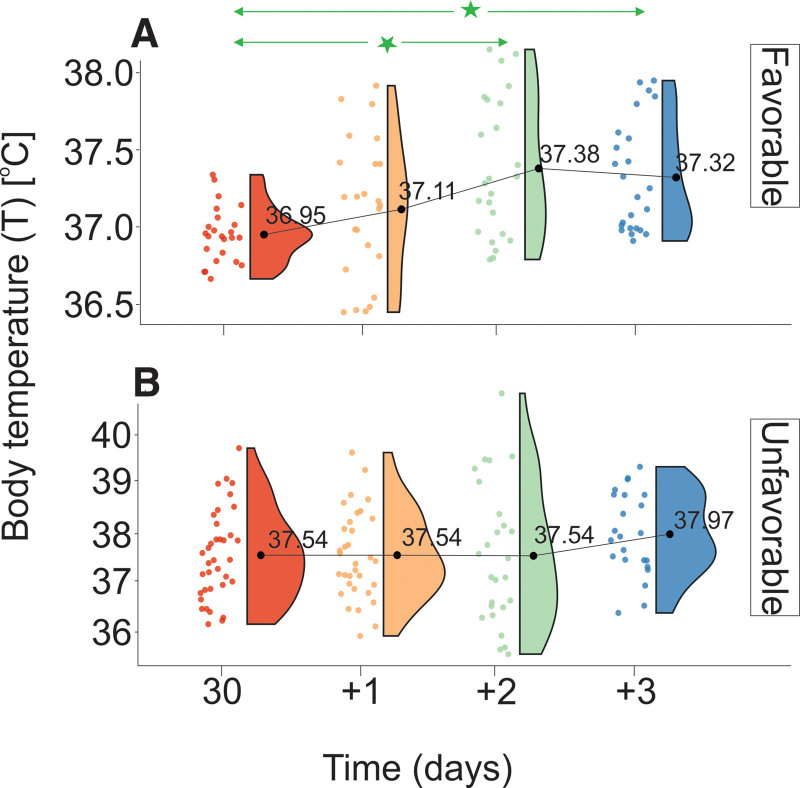
Changes in *T* stratified by GOS levels evaluated on discharge from the Department of Neurosurgery. (A) Favorable outcome group and (B) unfavorable outcome group. The vertical size of a cloud refers to 95% CI at a specific time point. The green arrow shows statistically significant differences in the “favorable” outcome group. CI = confidence interval, GOS = Gosling Outcome Scale, *T* = body temperature. ^*^P < .01.

The stratification scheme led to the post hoc assignment of 36 and 24 patients to UG and FG, respectively. The average age of the patients in the UG was 49 years (range 20–72) and that of the FG was 48 years (range 24–75). The standard reference range was 0.05 to 0.23 µg/L, 30.8 to 73.17 cm/s, and 0.62 to 1.13 for the S100B, *V*_mean_, and PI levels, respectively. Changes in S100B, *V*_mean_, PI, ICP, and *T* levels of the S100B group (measured at 30, +2, +3, and +4 days after hospital admission) are presented in Tables 1A and 1B. Differences between UG and FG at a specific time point for the levels of S100B, *V*_mean_, PI, ICP, and *T* are provided in Table 2. A graphical representation of the changes in S100B, *V*_mean_, PI, ICP, and *T* levels stratified by outcome groups is shown in Figures 2A and B–6A and B.

**Table 1A:** Descriptive statistics of S-100b, *V*_mean_, PI, ICP, and *T* levels as a function of hospitalization time in the UG group.

Parameter	Time (d)	Mean	SD	Median	Min	Max
S-100b	30	6.45	4.77	4.71	1.67	19.41
	31	5.23	3.43	4.42	1.14	16.67
	32	4.47	3.83	3.45	0.93	20.38
	33	3.74	3.15	3.25	0.79	15.91
*V*_mean_	30	27.15	8.18	25.44	5.18	42.03
	31	34.00	13.96	31.59	6.04	60.02
	32	34.64	26.18	33.75	5.06	118.46
	33	38.97	31.57	35.83	6.22	142.46
PI	30	3.46	1.04	3.25	1.85	7.08
	31	2.92	1.03	2.96	1.41	5.80
	32	2.98	1.23	2.86	1.11	6.61
	33	2.95	1.30	2.59	1.05	6.76
ICP	30	46.23	13.44	46.12	27.01	86.71
	31	33.64	13.58	35.61	11.12	66.52
	32	34.59	15.32	29.84	9.18	80.05
	33	34.45	15.34	37.2	7.21	60.74
*T*	30	37.54	0.92	37.45	36.13	39.73
	31	37.54	0.90	37.42	35.89	39.64
	32	37.53	1.43	37.29	35.52	40.85
	33	37.97	0.85	37.92	36.36	39.35

ICP = intracranial pressure, PI = pulsatility index, SD = standard deviation, *T* = body temperature, UG = unfavorable group, *V*_mean_ = mean maximum velocity.

**Table 1B:** Descriptive statistics of S-100b, *V*_mean_, PI, ICP, and *T* levels as a function of hospitalization time in the FG.

Parameter	Time (d)	Mean	SD	Median	Min	Max
S-100b	30	1.40	0.48	1.37	0.74	2.73
	31	1.14	0.45	1.00	0.48	1.9
	32	1.19	0.48	1.24	0.39	2.07
	33	0.83	0.36	0.85	0.14	1.61
*V*_mean_	30	43.92	8.58	42.76	31.68	65.52
	31	51.95	13.72	47.71	36.13	83.67
	32	50.12	11.79	49.74	29.02	73.04
	33	61.27	10.73	61.24	39.49	87.87
PI	30	2.04	0.39	2.06	1.23	2.87
	31	1.66	0.41	1.57	1.09	2.92
	32	1.61	0.47	1.57	0.95	2.97
	33	1.31	0.44	1.30	0.64	2.51
ICP	30	26.15	6.27	27.15	13.59	38.44
	31	19.42	4.49	18.54	11.27	27.88
	32	17.95	5.75	17.35	10.26	33.95
	33	14	4.58	13.19	7.09	23.51
*T*	30	36.95	0.18	36.94	36.67	37.34
	31	37.12	0.45	37.16	36.45	37.92
	32	37.38	0.45	37.3	36.79	38.15
	33	37.32	0.36	37.22	36.91	37.95

FG = favorable group, ICP = intracranial pressure, PI = pulsatility index, SD = standard deviation, *T* = body temperature, *V*_mean_ = mean maximum velocity.

**Table 2:** Statistical differences between means of analogous merriments for FG and UG.

Parameter	Time (d)	FG mean	UG mean	*P < *.01
S-100b	30	1.40	6.45	*
	31	1.14	5.23	*
	32	1.19	4.47	*
	33	0.84	3.74	*
*V*_mean_	30	43.92	27.15	*
	31	51.95	34.00	*
	32	50.12	34.64	
	33	61.45	38.97	
PI	30	2.04	3.46	*
	31	1.66	2.92	*
	32	1.61	2.98	*
	33	1.31	2.95	*
ICP	30	26.15	46.23	*
	31	19.42	33.64	*
	32	17.95	34.59	*
	33	14.11	34.45	*
*T*	30	36.95	37.54	*
	31	37.12	37.54	*
	32	37.38	37.53	
	33	37.34	37.97	*

FG = favorable group, ICP = intracranial pressure, PI = pulsatility index, *T* = body temperature, UG = unfavorable group, *V*_mean_ = mean maximum velocity.

* *P* < .01.

Analysis of normality unfolded different, in terms of normality, distributions of studied samples. This observation led to employment of bootstrap test for differences in the means. Such an approach allowed to overcome challenges of nonparametric statistics. Moreover, due to nonparametric nature of sample distribution, nonparametric factorial analysis of variance had to be employed to elucidate meaningful statistics.

No statistically significant time-dependent differences in S100B concentration were found in UG. However, a significant decrease in serum S100B levels was determined between 30 and +3 days in the FG measurements. The respective data are compiled in Tables 1A and 1B and Figure 2. The S100B levels of the UG patients were significantly higher than those of the FG at all measurement time points. The respective data are provided in Table 2. The difference in the S100B decrease velocity between the UG and the FG showed a relative decrease of 0.19, with velocities *V*_S100B_UG_ = 0.007 g/L/h and *V*_S100B_FG_ = 0.037g/L/h for the UG and FG, respectively.

An analysis of the data provided in Tables 1A and 1B and Figure 3 shows that the *V*_mean_ levels of the patients in the UG are characterized by a statistically lower *V*_mean_ than those of the FG 30 and 31 days after hospital admission. Statistically significant differences in *V*_mean_ levels were observed between 30 and +3 days in FG. There were no statistically significant differences in mean *V*_mean_ levels in the UG. The relative difference in *V*_mean_ levels between the UG and the FG was 1.4 (*V*_mean_UG_ = 0.21 cm/s/h and *V*_mean___FG_ = 0.15 cm/s/h).

Changes in PI levels are presented in Tables 1A and 1B and Figure 4. PI levels of UG patients were significantly higher than those of FG (Table 2). FG was defined by a decrease in PI (*V*_PI_UG_ = 0.14/day) and the lack of statistically significant differences between consecutive measurements. UG was defined by the lack of significant differences between the measurements, and the rate of decrease in PI was *V*_PI_FG _= 0.22/day. The relative ratio of the rate of decrease in PI between the UG and FG was $\frac{V_{PI\_UG}}{V_{PI\_FG}}=\frac{-0.006}{-0.009}=0.65$.

Changes in ICP levels are provided in Tables 1A and 1B and Figure 5. Differences in mean ICP levels between FG and UG are summarized in Table 2. ICP levels in the FG were significantly higher at all measurement time points than those in the UG (Table 2). UG was defined by a decrease rate of *V*_ICP_UG_ = 0.14 mm Hg/h, and FG was defined by a decrease rate of *V*_ICP_FG_ = 0.16 mm Hg/h. The relative ratio of changes in ICP levels between UG and FG was 0.88 mm Hg/h.

Data analysis provided in Tables 1A and 1B and Figure 6 shows that *T* levels of patients in UG are limited by statistically higher values than those of FG at 30, 31, and 33 days after hospital admission. Statistically significant differences in *T* levels were observed between 30 (+2) and 30 (+3) days in FG. There were no statistically significant differences in *T* levels in UG. The relative difference in the rate of increase in *T* levels between the UG and FG was 1.2 (*V*_T _ UG_ = 0.14°C/day and *V*_T_ __ FG_ = 0.12°C/day).

## 4. Discussion

Among the techniques used to predict the results of TBI and CBI treatment are mathematical models such as the International Mission for Prognosis and Clinical Trials in Traumatic Brain Injury^50^ and Corticosteroid Randomization after Significant Head Injury.^36^ However, they are of poor precision at the individual patient level.

In the search for prognostic methods for TBI, studies of biological markers were carried out. However, most of the studies conducted so far lack good discriminatory capacity.^51–59^

A new biomarker has recently been proposed for the diagnosis and prediction of the outcome of the treatment of TBI and CBI: the S100B protein.^60,61^ Furthermore, analysis of CBF velocity, particularly *V*_mean_ and PI, is derived through the TCD examination and can also be used in the diagnosis and prediction of the outcome of the treatment of TBI and CBI.^42^ A recent study has also shown that monitoring ICP levels influences treatment methods and can reduce mortality in people with severe TBI.^62^ *T* measurement can also benefit the treatment of TBI.^63^

This study attempted to advance knowledge of the applicability of specific markers and design a new diagnostic method and prediction of the outcome of TBI treatment. Therefore, a simultaneous analysis of changes in S100B, *V*_mean_, PI, ICP, and *T* levels was performed in patients with CBI stratified by treatment outcomes. To our knowledge, this is the first attempt to combine these parameters for the diagnosis and prediction of results.

Additionally, the standard reference range for S100B, *V*_mean_, and PI levels was obtained during this study.

The reference range for the standard S100B concentration established in this study was 0.02 to 0.23 µg/L. This result differs from the previously proposed concentration, 0.02 to 0.15 μg/L.^64^ The mean of the reference range was 0.1 µg/L, which differs from the previously reported value for Caucasians (0.07 µg/L)^65^ and indicates S100B levels in the range of the Asian population. Furthermore, it is similar to the mean value reported for Asians (0.11 µg/L). Since S100B levels appear to depend on age^66,67^ and sex,^67^ the observed difference may be the product of different male-to-female ratios and different mean ages of the study sample. This study shows that both treatment outcome groups (i.e., FG and UG) are defined by S100B levels significantly greater than the normal range. However, the levels observed in the UG are 4.5 times higher than those in the FG. Furthermore, assuming a continuous decrease in S100B levels at the rates observed for UG and FG (*V*_S100B_UG_ = 0.007 g/L/h and *V*_S100B_FG_ = −0.037 µg/L/h), the additional time required to reach the standard range would be 50.0 and 16.5 hours for UG and FG, respectively. This observation indicates that recovery of normal levels of S100B is crucial for the results of CBI and TBI treatment.

In this study, the reference range for the *V*_mean_ was 30.8 to 73.17 cm/s, while previous studies reported a range of 30 to 60 cm/s.^42^ Although the lower limits are similar, the upper limits reported in this study are higher. In the current state, it is difficult to identify what is causing this difference. Among the possible factors may be the age of the study sample, the male-to-female ratio, differences in mean arterial pressure, and differences in *p*CO_2_.^68,69^

Analysis of changes in *V*_mean_ as a function of treatment results helped determine levels in the third quartile of the normal reference range in the FG and in the first quartile of the normal reference range in the UG. Furthermore, the rate of increase in the *V*_mean_ levels that define the UG was 1.4 times greater than that observed in the FG: *V*_mean_UG_ = 0.21 cm/s/h and *V*_mean___FG_ = 0.15 cm/s/h. The results presented by van Santbrink et al,^70^ in conjunction with the results of this study, confirmed that an unsatisfactory outcome of TBI treatment is defined by reducing the velocity of CBF. This study also confirmed previous data on the relationship between mean blood flow velocity and the outcome of TBI treatment (44 and 36 cm/s for good and poor outcomes, respectively^71^), showing that FG was defined by overall *V*_mean_ levels greater than those observed in UG (54.5 and 33.69 cm/s for FG and UG, respectively).

In this study, the reference range for PI levels was 0.62 to 1.13. The previously reported reference range was 0.5 to 1.19.^43^ According to a previous study, a PI level < 0.5 may indicate arteriovenous malformation^72^ or proximal stenosis occlusion, while a PI level > 1.199 may indicate constriction or distal occlusion.^73^ PI ≤ 1.0 defines patients with GOS scores of 4 to 5,^71^ while patients with a PI level ≥ 1.56 are at risk of poor treatment results.^71^ However, others reported a PI level of 1.25 as the threshold that defines the results of TBI treatment.^74^ This study revealed a distinct difference in time dependent PI change (−0.14 and 0.22/day for UG and FG, respectively) and a significantly lower PI level in FG compared to UG. Furthermore, this study confirmed the ranges but not absolute values of the previous study (good result = 1.00 and poor result = 1.56^71^) through the relations of the overall PI means 1.52 and 2.95 for FG and UG, respectively. Therefore, in this study, the result that defines the poor outcome in the previous study refers to a favorable outcome. Since both studies used an analogous GOS stratification model, that is, scores 1 to 3 (1 = *death*, 2 = *vegetative state*, and 3 = *severe disability*) were considered “poor” and unfavorable outcomes, and scores 4 to 5 (4 = *moderate disability* and 5 = *complete recovery* or *correct outcome*) were considered “good” and favorable outcomes; the etiology of the observed differences is unknown.

Elevation of ICP levels is accompanied by an incidence of brain injury^75^ that may be caused, among others, by reduced CPP. Furthermore, prolonged elevated ICP levels can lead to cerebral ischemia, brain herniation, and death. Unfortunately, due to the different methodological approaches used, the cross-correlation of ICP levels between patients from different countries is of poor diagnostic quality.^76^ Although the generally adopted ICP threshold is equal to 20 to 25 mm Hg,^77,78^ applying the previously reported linear relationship between PI and ICP (ICP = 11.1 × PI – 1.13^79^) unfolded ICP in the range of 5.45 to 11.11 mm Hg. However, this equation provided a poor relationship between ICP and PI in UG and FG stratified by measurement time points. In general, patients in UG were defined by ICP in the range (ICP_r_UG_) 7.21 to 86.71 mm Hg, while those in FG (ICP_r_FG_) were defined by ICP 7.09 to 38.44 mm Hg. Therefore, ICPr_UGICPr_FG results in an ICP threshold of 39 mm Hg, indicating a very high risk of unsuccessful treatment in patients with ICP 39 mm Hg. This observation confirms the body of reports that implies that an increase in ICP levels increases the odds of unsuccessful treatment outcomes.^80^ This study also revealed a continuous decrease in ICP levels at a rate of 0.14 and 0.16 mm Hg/h for UG and FG, respectively. Furthermore, the results of our study differed slightly from the previous study,^71^ reporting a mean ICP of a good result of 15 mm Hg and a poor result of 30 mm Hg, providing values much higher for FG and UG (17.16 and 34.22, respectively). However, the UG is defined by 2 times the ICP levels observed in the FG. Furthermore, this study confirmed previous reports indicating that ICP levels > 25 to 30 define poor results from TBI treatment.^81,82^

Currently, there is a lack of cross-correlation studies between *T* and the prognosis of treatment for CBI. Although a healthy brain is resistant to fever compared to the injured brain, prolonged exposure to fever can result in infarcted brain cells.^83^ Therefore, elevated brain temperature has been shown to result in an increased risk of unfavorable outcomes of CBI treatment.^84,85^ Since core *T* and an increase in cerebral metabolism of 1°C increase cerebral metabolism by 7% to 13%,^28,63^ both groups are defined by a small increase in cerebral metabolism of the order of 3 to 6 and 2.5% to 5.0% for UG and FG, respectively. Furthermore, the previous study^83^ showed the lack of direct correlation between body and brain temperatures in disease. However, our study revealed significantly higher *T* in the UG than in the FG, indicating *T* as the prediction parameter of the outcome of treatment. Currently, the etiology of this phenomenon is unknown. It may be caused by the release of factors that increase *T*, such as cytokines,^86^, interleukins,^87^ and white blood cells.^88^ However, taking into account the observation that brain temperature in head trauma patients is on average 0.22°C higher than *T*^89,90^ and that normal *T* is < 37.5°C,^91^ UG patients are defined by the detrimental increase in *T* that was reflected in treatment outcomes.

**Figure 7.**
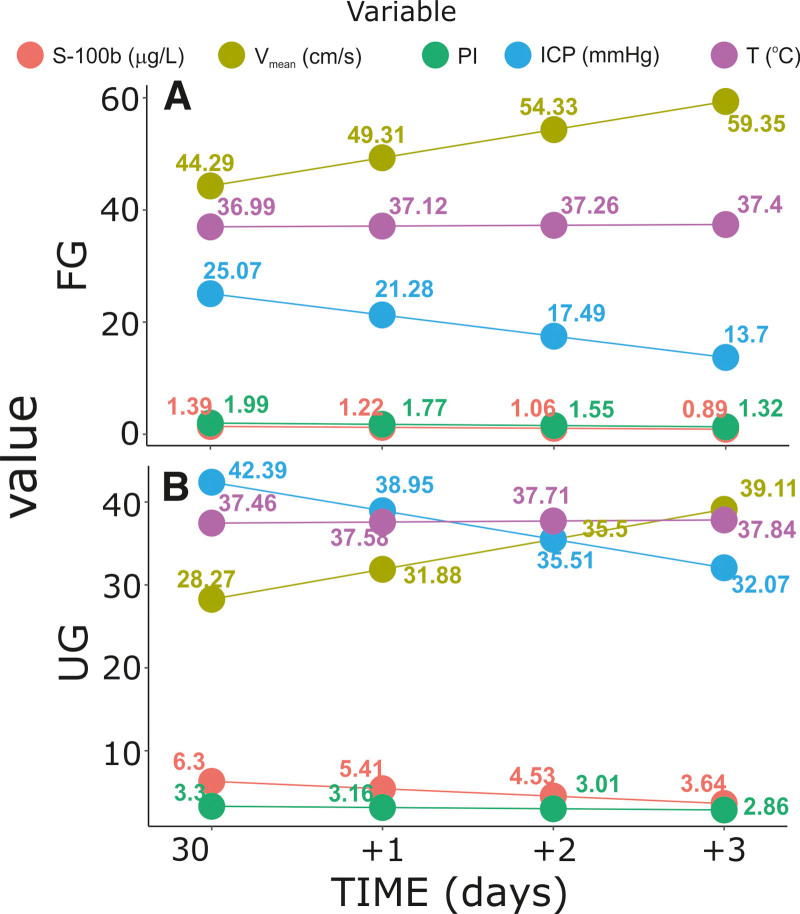
Relative changes in S100B, *V*_mean_, PI, ICP, and *T* levels stratified by GOS levels evaluated on discharge from the Department of Neurosurgery. (A) Favorable outcome group and (B) unfavorable outcome group. The number of measurement time points refers to the mean level. The curves are the product of linear regression models. GOS = Gosling Outcome Scale, ICP = intracranial pressure, PI = pulsatility index, *T* = body temperature, *V*_mean_ = time-averaged mean maximum velocity.

The analysis of time-dependent changes revealed the following relations between the parameters studied in FG and UG, respectively: (1) an apparent increase in *V*_mean_ levels; a decrease in levels of S100B, PI, and ICP levels; and a virtually constant *T* (Fig. 7A); (2) an increase in *V*_mean_ levels; a decrease in levels of ICP, S100B, and PI levels; and constant *T* (Fig. 7B). Although both groups are defined by the analogous direction of changes in the parameters studied, it is clear that UG is defined by higher levels of ICP, *T*, S100B, and PI and lower levels of *V*mean.

In conclusion, it can be stated that S100B < 3 mg/L, PI < 2.86, ICP > 25 mm Hg, and *V*_mean_ > 40 cm/s defined the group of favorable outcomes. Although more studies are required to design a robust outcome prediction model, the data provided may already serve as an indicator of the results of TBI treatment.

This presented study in a novel combination, not published elsewhere, of multiparameter analysis allowing to observe cross-correlation among the studied parameters; S100B, PI, ICP, and *V*_mean_ allowing for more accurate proetid ion of treatment outcome in clinical environment.

Nevertheless, to improve the statistical power of the studied correlations, we envisage to extend the number of subjects studied as well as introduce a multicenter study.

## Author contributions

**Conceptualization:** Sebastian Dzierzęcki, Mirosław Ząbek, Ryszard Tomasiuk.

**Data curation:** Sebastian Dzierzęcki, Gabriela Zapolska.

**Formal analysis:** Sebastian Dzierzęcki, Ryszard Tomasiuk.

**Funding acquisition:** Sebastian Dzierzęcki.

**Writing – original draft:** Gabriela Zapolska, Ryszard Tomasiuk.
